# Potential Therapeutic Candidates for Age-Related Macular Degeneration (AMD)

**DOI:** 10.3390/cells10092483

**Published:** 2021-09-19

**Authors:** Sonali Nashine

**Affiliations:** Department of Ophthalmology, University of California Irvine, Irvine, CA 92697, USA; snashine@uci.edu

**Keywords:** age-related macular degeneration (AMD), retina, AMD therapeutics

## Abstract

Aging contributes to the risk of development of ocular diseases including, but not limited to, Age-related Macular Degeneration (AMD) that is a leading cause of blindness in the United States as well as worldwide. Retinal aging, that contributes to AMD pathogenesis, is characterized by accumulation of drusen deposits, alteration in the composition of Bruch’s membrane and extracellular matrix, vascular inflammation and dysregulation, mitochondrial dysfunction, and accumulation of reactive oxygen species (ROS), and subsequent retinal pigment epithelium (RPE) cell senescence. Since there are limited options available for the prophylaxis and treatment of AMD, new therapeutic interventions are constantly being looked into to identify new therapeutic targets for AMD. This review article discusses the potential candidates for AMD therapy and their known mechanisms of cytoprotection in AMD. These target therapeutic candidates include APE/REF-1, MRZ-99030, Ciliary NeuroTrophic Factor (CNTF), RAP1 GTPase, Celecoxib, and SS-31/Elamipretide.

## 1. Introduction

Aging manifests at multiple molecular and physiological levels and contributes to the etiology of several chronic neurodegenerative and ocular diseases in humans. In normal healthy aging, due to environmental insults and natural cellular senescence, genomic stability and biological functions are compromised resulting in genetic lesions. The major molecular and cellular hallmarks of normal aging include spontaneous mutations in nuclear and mitochondrial DNA, mitochondrial dysfunction, decline in DNA repair mechanisms, shortening of telomeres, altered epigenetic patterns including chromosomal remodeling, loss of cellular protein homeostasis, altered endocrine homeostasis, modified cell signaling, excessive melanin production, and accumulation of lipofuscin and melanosomes [1,2].

In the retina, aging also causes retinal pigment epithelium (RPE) cell senescence, accumulation of metabolic debris and protein aggregates, thickening of Bruch’s membrane, and alteration in the composition of Bruch’s membrane and extracellular matrix. In eyes of normal, young individuals, i.e., less than 45 years of age, the RPE cells are uniformly organized as a monolayer of polygonal (mostly hexagonal) cells with sharp vertices, at the fovea and periphery. Aging leads to reduction in the pattern of hexagonal cells, resulting in enlarged, irregular pleomorphism of cells to cover the Bruch’s membrane. However, despite irregular geometry, the overall number of RPE cells at the posterior pole remains constant in older retinas [3,4]. With aging, the choroid undergoes thinning, choriocapillary density is reduced, choroidal flow and perfusion is decreased, and vascular inflammation is observed within the retina and the choroid. Retinal vascular inflammation involves morphological changes in microglial cells that migrate into the subretinal space, cause upregulation of inflammatory cytokines, and loss of vascular smooth muscle cell from retinal arterioles [5,6].

When an individual approaches 40 years of age, the lens proteins are modified so the lens becomes less flexible and loses its ability to focus on near images. This condition is referred to as Presbyopia that can be treated with the use of ‘reading glasses’ [7]. As people approach their sixth decade, the lens proteins become brunescent, loose transparency and cataracts are formed. Fortunately, cataract removal with placement of an intraocular artificial lens is a common surgery that has very high success rates. Another impediment to vision is that the pupils become smaller, react sluggishly to light, and dilate relatively slowly in the dark, due to weakening of muscles that regulate the pupil.

Although aging is a principal risk factor for many eye diseases, it may not necessarily lead to ocular disease pathology. Age-related alterations, environmental stressors, and genetic signatures are risk factors for Age-related Macular Degeneration (AMD), which is a chronic, debilitating, ophthalmic disease that causes visual deterioration in the elderly. The risk of AMD-associated vision loss increases with age and is a primary cause of legal blindness in the U.S. affecting over 14 percent of white Americans aged 80 and older, per the NEI (National Eye Institute) data. Caucasian Americans have the greatest likelihood of developing AMD, accounting for 89%, whereas black and Hispanic American populations each accounted for 4 percent of AMD cases. NEI’s statistical projection indicates that as the proportion of U.S. population over 65 years of age increases, the prevalence of AMD cases is expected to rise from 2.07 million to 5.44 million by 2050.

The molecular underpinning of transition from normal aging processes to pathological AMD is primarily attributed to vascular inflammation and dysregulation, mitochondrial damage and accumulation of reactive oxygen species (ROS) along with RPE cell senescence. AMD is a multifactorial disease, i.e., various complex etiologies including genetic and lifestyle factors underlie its predisposition, development, and severity. In early AMD, the RPE cytoskeleton is altered, causing loss of normal regular polygonal shape, RPE cell death and hypopigmentation of the retina. As the RPE cytoskeleton is altered, cells enlarge and fuse causing focal splitting, fragmentation, thickening and the appearance of intracellular stress fibers [8,9]. Accumulation of pathological molecular debris and drusen deposition between RPE cells and the choroid is referred to as early dry AMD and, when it becomes severe with loss of both RPE cells and photoreceptors, it is called geographic atrophy. Severe AMD is characterized by irreversible damage to the macula, a cone photoreceptor-dominated region approximately 5.5 mm in diameter, which is responsible for central vision and high-resolution visual acuity. In AMD pathology, the degeneration of cone photoreceptor cells in the fovea of the macula results in thinning of macula and blurring of central vision. In approximately 15% of cases, there is an altered balance of pro-angiogenic and anti-angiogenic factors that cause wet/neovascular AMD [10]. Choroidal neovascularization (CNV) observed in wet macular degeneration is often triggered by excessive production of VEGF (Vascular Endothelial Growth Factor) and subsequent breakdown of the blood–ocular barrier. CNV involves formation of new, abnormal blood vessels that originate in the choroid and grow into the subretinal space leading to influx of fluids and macromolecules from the blood into the retina, thereby affecting central vision [11]. To maintain cellular and physiological homeostasis, retinal pigment epithelium (RPE) barriers undergo continuous assembly and turnover. Ocular damage caused by intraocular inflammation, surgical or non-surgical traumas and intraocular tumors results in cytokine release, increased VEGF production, and immune response activation leading to the breakdown of the blood–ocular barrier. The blood–ocular barrier, which is composed of the Blood–Aqueous Barrier (BAB) and the Blood–Retinal Barrier (BRB), is crucial to maintaining ocular physiological homeostasis and protecting the eye [12]. The BRB tightly regulates the flux of solutes, fluids, macromolecules, and ions into and out of the retina. The BRB consists of the outer barrier formed by a monolayer of RPE cells connected by tight junctions (Zonulae Occludentes), and the inner barrier which is established by tight junctions between the endothelial cells of retinal vessels [12,13].

Fortunately, the wet form of AMD can be treated by intraocular injections of anti-VEGF drugs that keep the neovascularization and retinal thickening at a minimum [14]. To date there is no treatment for the dry form of AMD that is increasing in occurrence and can also significantly decrease visual acuity. Therefore, research focused on dry AMD therapy is on the rise and in addition to a variety of treatment options including steroids, antioxidants, several neuroprotective agents have been tested in vitro and in vivo to delay AMD associated retinal degeneration. Basic research has made significant advances in our understanding of mechanisms underlying the action of bioactive molecules for treatment of AMD. Of the wide variety of therapeutic agents used to treat AMD, this review discusses the latest and emerging candidates for AMD therapy and highlights their mechanism of cytoprotection in AMD.

## 2. Overview of Potential Therapeutic Candidates for AMD

### 2.1. APE/REF-1

Age-related oxidative DNA damage, impaired DNA repair mechanisms, accumulation of reactive oxygen species (ROS), and subsequent cytotoxicity are contributory to retinal damage in AMD [15]. Previous studies have associated AMD mitochondrial and cellular damage to significant upregulation of mitochondrial superoxide and oxidative stress markers [16,17]. Therefore, various approaches that aim to lower oxidative stress have been tested as prophylactics and therapeutics for AMD in in vitro and in vivo models [18,19,20,21].

The human APE/REF-1, i.e., Apurinic/Apyrimidinic Endonuclease/Redox Effector Factor-1 is a ubiquitously expressed predominant AP (Apurinic/apyrimidinic) endonuclease in humans. This enzyme is found in the nucleus as well as the cytoplasm. It is a multifunctional enzyme that maintains cellular homeostasis, and also functions in DNA repair and redox (reduction-oxidation) regulation in response to oxidative stress and DNA damage. The *C*-terminal domain of APE/REF-1 possesses the DNA repair Endonuclease function and the *N*-terminal domain contributes to its redox activity. The human APE/REF-1 protein (36.5 kDa) is encoded by a ~3 kb gene on chromosome 14 bands q11.2–12. Both endogenous stimuli, (peroxisomes and mitochondria, along with increased levels of calcium, amyloid-β peptide, α-synuclein, Tau protein, hypoxia, ischemia-reperfusion injury); and exogenous stimuli (pollutants, smoke, xenobiotics, UV radiation) contribute to the activation of APE/REF-1. Abasic AP sites are formed in the DNA either spontaneously due to free radicals or by DNA glycosylases in the base excision repair pathway. During the processing of oxidatively damaged mutagenic DNA in the base excision repair pathway, AP endonucleases hydrolyze the phosphodiester backbone of the AP site to repair the damaged nucleotides in DNA. The repair activities of APE/REF-1 include an endonuclease and 3′- diesterase activities. During this DNA repair process, APE-1 interacts with several DNA repair pathway proteins, such as OGG1 (a DNA glycosylase), XRCC1, FEN1, GADDα, PCNA, PARP1, GAPDH, DNA Polymerase β, and DNA ligases. This DNA repair function of APE/REF-1 is critical in cellular protection since the unrepaired AP sites in the DNA lead to DNA strand breaks, apoptotic cell death and cytotoxicity. Repair and removal of AP sites occurs in damaged mtDNA as well and the mitochondrial AP endonuclease activity plays a role in eliminating damaged mitochondrial genomes from the gene pool.

The repair and redox properties of APE/REF-1 function independently of each other and are equally crucial. The redox potential of APE/REF-1 is unique to mammals. In APE/REF-1, three of the seven conserved cysteine residues namely Cys65, Cys93, and Cys99 primarily contribute to the redox function. APE/REF-1 catalyzes thiol-mediated redox reactions in which the target proteins, which are mostly transcription factors, are reduced, and the redox factor is oxidized in the process. The APE/REF-1 redox factor regulates the activities of various transcription factors including AP-1, NF-κB, p53, CREB, c-Jun, c-Fos, HIF-1α, and PAX. REF-1 directly associates with Thioredoxin (TRX) and stimulates the DNA-binding activity of AP-1 to facilitate redox regulation [22]. It has been shown that, by promoting NF-κB activity, the REF-1 mediates pro-survival signaling in vascular endothelium [23].

**Figure 1:**
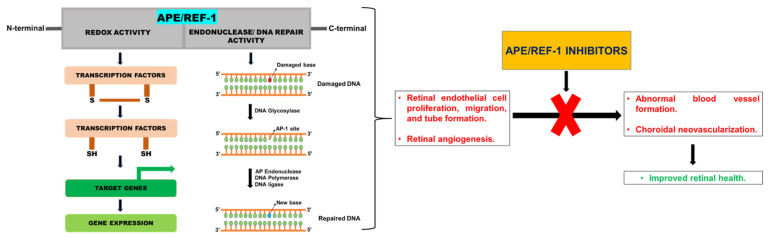
APE/REF-1 inhibitors’ mechanism of action. APE/REF-1 possesses both redox activity and DNA repair activity via which it contributes to retinal angiogenesis, endothelial cell proliferation, migration, and tube formation. Addition of APE/REF-1 inhibitors blocks abnormal blood vessel formation and choroidal neovascularization, thereby improving retinal health.

In the eye, APE/REF-1 redox activity is needed for retinal endothelial cell proliferation, migration, and tube formation. Since APE/REF-1 has been shown to contribute to retinal angiogenesis, it is therefore a potential target for AMD therapy. With neovascularization being a major contributor of AMD pathology, the small molecule inhibitors of APE/REF-1 can potentially block abnormal vessel formation. Systemic administration of two newly discovered REF-1 inhibitors, APX2009 and APX2014, blocked REF-1 redox signaling and attenuated CNV in vivo [24]. In another study, E3330—a small molecule inhibitor, reduced CNV both in vitro and in vivo by suppressing APE/REF-1 redox function [25] (Figure 1).

### 2.2. MRZ-99030

Drusen, deposits of extracellular material between the RPE and Bruch’s membrane, are a hallmark of AMD. With aging and progression of AMD, drusen increase in size and numbers, become more confluent, cause RPE and photoreceptor degeneration, and damage the choroidal vasculature [26,27,28,29]. Amyloid-β is a primary constituent of drusen and is a biomarker for AMD. It accumulates in the sub-RPE space and triggers pro-inflammatory, pro-angiogenic, and complement pathways, leading to the neurotoxicity observed in AMD etiology [30,31]. Amyloid-β also impairs the microtubule-associated protein 2 (MAP-2), thereby affecting synaptic plasticity [32]. Increased retinal amyloid-β burden above the normal physiological levels is observed in aged and diseased AMD eyes. Of the various forms of amyloid-β embedded in drusen deposits, 1–42 amino acid amyloid-β is found to be more neurotoxic due to its higher tendency of oligomerize [33,34]. Overproduction of monomers of amyloid-β fibrils causes misfolding and the monomers spontaneously aggregate to form toxic oligomers, contributing substantially to AMD progression. Accumulated amyloid-β aggregates cause increased VEGF expression and eventually contribute to the atrophic and neovascular pathologies in AMD [35,36,37]. Amyloid-β peptides are widely used to mimic the AMD-like pathology in an attempt to understand the mechanisms and screen potential candidate drugs that could rescue from amyloid-β-induced toxicity. For instance, to create an AMD model, amyloid-β peptides were overexpressed in RPE cells resulting in drusen-like deposits and AMD-like retinal pathophysiology [38]. Therefore, a plethora of studies have focused on suppressing the accumulation of amyloid-β aggregates to prevent retinal toxicity and achieve retinal neuroprotection in AMD.

Since normal synaptic functioning in the eye requires amyloid-β in its monomeric form, preventing amyloid-β from oligomerization is a logical approach to inhibit amyloid-β-induced toxicity. To this end, Merz Pharma GmbH & Co. KGaA (Frankfurt, Germany) developed a small molecule drug, MRZ-99030 [39] that is an inhibitor of amyloid-β aggregation. MRZ-99030 is a dipeptide with a molecular weight of 289 Da and the chemical formula: NH2-d-Trp-Aib-OH/2-(2-Amino-3-(1H-indol-3-yl)-propionylamino)-2-methylpropionic acid). MRZ-99030 reduces the amount of toxic soluble amyloid-β_1-42_ oligomers by promoting the formation of innocuous, globular, non-β-sheet, amorphous aggregates of amyloid-β_1-42_. MRZ-99030 is composed of an aromatic moiety and β-sheet breaker species and does not obstruct protein–protein interactions between amyloid-β monomers; however, it interferes with aromatic stacking to suppress the formation of toxic amyloidogenic fibrillar aggregates with β-sheet structure, thereby switching to a non-amyloidogenic pathway and providing sustained prevention of amyloid-neurotoxicity [40]. MRZ-99030 also reduces the number of apoptotic cells and attenuates amyloid-β toxicity significantly by formation of amyloid-β_1-42_ assemblies that are benign to the retina [41]. Its unique mechanism of action, good bioavailability and tolerability as observed in in vitro and in vivo models, and its demonstrated safety in Phase 1 clinical studies, makes MRZ-99030 a potential candidate for AMD therapy.

An important feature of MRZ-99030 is a self-propagation mechanism, whereby the “blobs” retain their ability to collect additional misfolded amyloid beta monomers, even in the absence of additional MRZ-99030 molecules. Galimedix Therapeutics, Inc. patented this novel “trigger effect” which results in sustained effects lasting far longer than the time for which a single administered dose has therapeutic retinal levels. This potentially allows for convenient, sustained benefit during the inter-treatment intervals for patients. Therefore, MRZ-99030 eye drops may prevent formation of toxic amyloid beta oligomer deposits, thereby clearing the system of these pathological factors.

**Figure 2:**
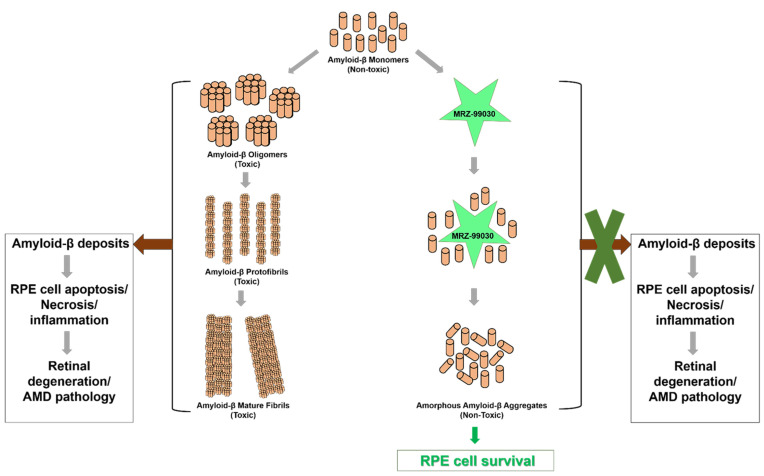
MRZ-99030′s mechanism of action. MRZ-99030 binds to amyloid-β monomers resulting in amorphous, non-toxic amyloid-β aggregates, thereby preventing their assembly into toxic mature amyloid-β fibrils. This in turn blocks the accumulation of amyloid-β deposits and prevents RPE cell degeneration and AMD pathogenesis.

Recently, Galimedix Therapeutics, Inc. developed GAL-101 i.e., novel small molecule eye drops, to delay the progression of dry AMD and glaucoma [42]. The route of administration is transscleral via the choroid. GAL-101 offers >90% neuroprotection in animal models and efficient retinal delivery in non-human primates. Phase 1 clinical trials with GAL-101 were completed successfully with no evidence of toxicity. Currently, GAL-101 is entering Phase 2 efficacy trials, designed to offer cellular neuroprotection and improved visual functions in patients with retinal diseases, including those with dry AMD (Figure 2).

### 2.3. Ciliary NeuroTrophic Factor (CNTF)

Ciliary Neurotrophic Factor (CNTF), a 22 kD protein, is a potent survival factor for neurons and belongs to the InterLeukin-6 (IL-6) family of neuropoietic cytokines. CNTF was first discovered in the chick eye where it exerts its neurotrophic activity in ciliary neurons [43]. CNTF provides significant neuroprotection of rod and cone photoreceptors [44]. The apical membrane of human fetal RPE cells expresses the CNTF-specific receptors and mediates downstream signal transduction including the JAK (Janus kinase)/STAT (Signal Transducer and Activator of Transcription) pathway, which enhances RPE survival. CNTF binds to the receptor CNTFα that is bound to gp130 (Glycoprotein 130) and LIFR (Leukemia Inhibitory Factor Receptor) receptors on the RPE cell membrane, to activate JAK1 and JAK2. Activated JAK proteins in turn mediate the phosphorylation of STAT3. This is followed by translocation of phosphorylated STAT3 proteins to the nucleus where they bind to the nuclear DNA and activate transcription of genes that subsequently increase human RPE cell survival, along with fluid absorption and transport across RPE cells. It also elevates the polarized secretion of neurotrophic factors and cytokines in cultured human fetal RPE cells in vitro, thereby preserving the health and integrity of RPE and neuroretina [45]. In stress conditions, such as oxidative stress and/or hydrogen peroxide injury, corneal endothelial (CE) cells express CNTF which in turn protects these cells [46]. CNTF, released from Schwann cells after nerve injury becomes available to injured neurons; CNTF enables and promotes the in vivo survival of adult motoneurons with lesions [47].

As demonstrated in several studies, delivery of neuroprotectants to the eye is tricky because the BRB prevents the passage of molecules from systemic circulation to neurosensory retina. Systemic and bolus administration of CNTF is short-lived with a plasma half-life of 2.9 min. Moreover, CNTF can be highly toxic causing adverse side effects [48,49,50]. CNTF delivery using eye drops presents limitations such as systemic absorption, enzymatic degradation, tear turn over, and crossing the corneal BAB and BRB. Therefore, it is necessary to develop a method that delivers the optimal dose of CNTF directly to the target site, in this case the eye. One approach to circumvent problems is by local, sustained delivery of CNTF to the retina to maximize efficacy and minimize the harmful side effects.

**Figure 3:**
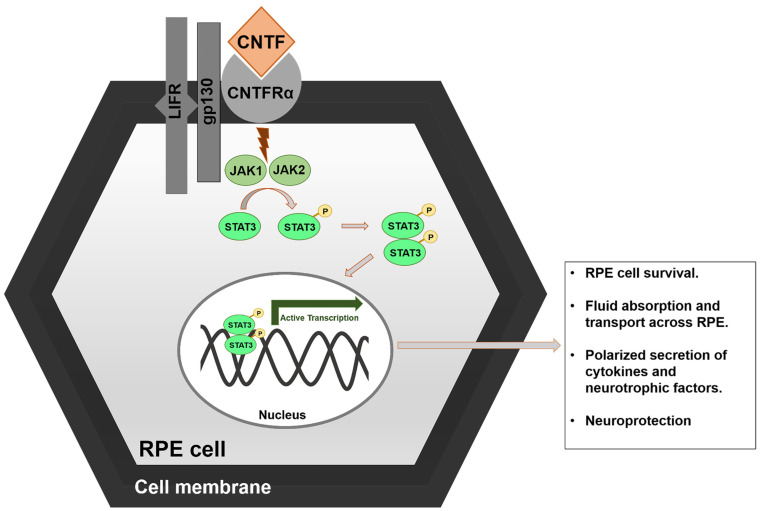
Ciliary NeuroTrophic Factor (CNTF)’s mechanism of action. CNTF binds to a trimeric receptor complex comprised of CNTFRα, gp130, and LIFR leading to its activation. Active CNTF results in phosphorylation of STAT3 which then activates transcription of target genes in the nucleus. This leads to RPE cell survival, fluid absorption and transport across the RPE, polarized secretion of cytokines and neutrophic factors, and subsequent neuroprotection.

To achieve this goal, encapsulated cell technology (ECT) was used to develop NT-501 implants (Renexus^®^, Neurotech Pharmaceuticals, Inc., Cumberland, RI, USA) that allowed efficiently controlled, sustained delivery of CNTF into the vitreous cavity of patients with atrophic macular degeneration. These implants offer the advantage of bypassing the BRB, minimal drug usage, causing less systemic exposure of the drug, and provide better control of disease. This retrospective study conclusively demonstrated that the NT-501 implant enables efficacious steady-state delivery of CNTF in the human eye over a 24-month implant period. A favorable pharmacokinetic and safety profile without systemic exposure supports the clinical development of the CNTF-secreting intraocular NT-501 implant for the treatment of chronic retinal degenerative diseases/conditions such as geographic atrophy/dry AMD and retinitis pigmentosa (ClinicalTrials.gov numbers, NCT00063765, NCT00447954, NCT00447980, NCT00447993) and may be further extended for the treatment of neovascular AMD [51] (Figure 3).

### 2.4. RAP1

Efficient regulation of angiogenesis requires effective functioning of tight junctions that serve as selectively permeable barriers that regulate the transepithelial diffusion of water, ions, and molecules between blood vessels and retina via the paracellular pathway. Tight junctions that possess transmembrane proteins such as Occludins, Claudins, and Junction adhesion molecule proteins are critical to maintaining cell integrity. Furthermore, adherens and tight junctions associate with the actin cytoskeleton to preserve junctional strength and function. Cell junctional regulation involves GTPases (Guanosine TriPhosphatases) which serve as signal transducers and molecular switch cycling between the GTP-bound active form and GDP-bound inactive form.

Ras-Associated Protein (RAP) belongs to the Ras family of cell-signaling proteins and plays a critical role in regulation of cell adherens junctions and cell differentiation [52]. RAP1 is a small GTPase that promotes junctional assembly, endothelial cell junction strengthening, [53] and inhibition of leukocyte transendothelial migration [54]. RNAi knockdown of RAP1 and RapGAP-mediated negative regulation of RAP1 caused loss of RAP1 protein function that resulted in compromised dynamic junctional reassembly kinetics, impaired RPE cell junction regulation, and enhanced choroidal endothelial cell transmigration in an in vitro choroial neovascularization model [55].

**Figure 4:**
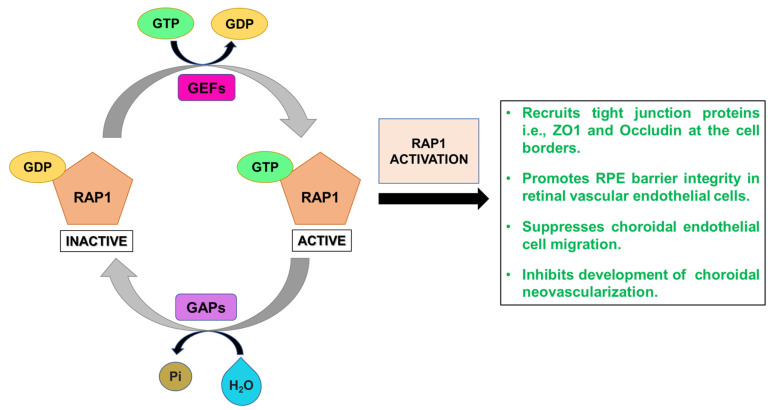
RAP1′s mechanism of action. Inactive RAP1 protein is converted to its active form by GEFs (Guanine nucleotide Exchange Factors) that activate monomeric RAP1 GTPase by stimulating the release of GDP (Guanosine DiPhosphate) to allow binding of GTP (Guanosine TriPhosphate), resulting in active RAP1. Active RAP1 recruits tight junction proteins, i.e., ZO1 and Occludin at the cell borders, promotes RPE barrier integrity in retinal vascular endothelial cells, suppresses choroidal endothelial cell migration, and inhibits development of choroidal neovascularization.

RAP1 exists in two homologous isoforms namely RAP1a and RAP1b which have 95% amino acid sequence homology, broad tissue distribution, and are not functionally redundant [56]. RAP1a and RAP1b isoforms are expressed in the RPE where they promote barrier integrity of RPE monolayers. While loss of *Rap1* is fatal, the knockout of one of the *Rap1* isoforms does not cause lethality. *Rap1b* plays a crucial role in maintenance of endothelial vasculature, [57] and is considered to be a potential antithrombotic therapeutic candidate because it regulates platelet function and plays a vital role in integrin signaling in platelets [54,55,56,57,58,59]. *Rap1a* and *Rap1b* participate in conformational activation and regulation of integrins in endothelial cells, and also contribute to angiogenic sprouting, adhesion, and migration [60]. Angiogenic factors rapidly enhance RAP1 activity in endothelial cells. RAP1 inhibits choroidal endothelial cell transmigration of the RPE cells which subsequently augments RPE barrier function [54]. RAP1 regulates vascular endothelial cadherin-mediated cell–cell adhesion [61], along with integrin-mediated and cadherin-mediated adhesions. Loss of Rap suppresses cell adhesion to both E-cadherin and Fibronectin. RAP1 most likely regulates E-cadherin homotypic adhesions via interaction with the actin cytoskeleton. It is speculated that AF-6 is a Rap-binding effector protein found at the adherens junctions and modulates cadherin–cadherin interactions. Furthermore, RAP1 signaling modulates integrin-mediated cell interactions [62] via changes in clustering and ligand affinity of integrins. RAPL, a Rap-binding protein, is involved in integrin cell adhesions [63] and plays a role in the angiogenic effects of RAP1 [60]. Hogan et al. demonstrated that Cdc42, a Rho small GTPase family member, acts downstream of RAP1 to form cadherin-mediated cell–cell contacts [63,64]. EPAC (exchange factor directly activated by cAMP) is a Rap guanine nucleotide exchange factor directly activated by cAMP. The cAMP effector protein mediates exchange of GDP for GTP and contributes to the activation of Rap [65]. In mammals, EPAC exists in two isoforms: EPAC1/RapGEF3 and EPAC2/RapGEF4 [66]. Activation of EPAC-RAP1 signaling recruits tight junction proteins at the cell borders, thereby promoting basal barrier properties in retinal vascular endothelial cells that form a part of the BRB. Moreover, the EPAC-RAP1 pathway inhibits RhoA GTPase and VEGF receptor signaling via the Ras/ERK (Extracellular-signal-Regulated Kinase) pathway and restores barrier integrity that has been compromised due to VEGF-induced or cytokine-induced permeability [67]. The endothelial barrier function is coordinated by cAMP-dependent pathways such as EPAC-RAP1 signaling and PKA (Protein Kinase A) signaling [68]. In a laser induced CNV rodent model, Li et al. demonstrated that activation of RAP1 using intravitreal injections of a cAMP analog, i.e., 8-CPT-cAMP (8-(4-Chlorophenylthio)adenosine 3’,5’-cyclic monophosphate), reduced the size of CNV lesions, decreased VEGF expression in RPE/choroid tissue, down-regulated NOX4 transcript and protein, and substantially reduced ROS levels in the RPE at CNV lesions. Moreover, RAP1 activation enhanced the expression of two tight junction proteins, i.e., ZO-1 (Zonula Occludens-1) and Occludin thereby promoting RPE barrier integrity and inhibiting CNV development in vivo [69]. Previous studies have demonstrated that inhibition of RAP1 activity by RAP1-knockdown in RPE monolayers compromised junctional integrity as evidenced by lowered transepithelial electrical resistance, enhanced transmigration of choroidal endothelial cells, mislocalization of cadherins and formation of gaps within the RPE monolayer [55]. Wang et al. demonstrated specific activation of RAP1a by subretinal injection of constitutively active RAP1a to RPE cells in mouse eyes, using an RPE-specific GFP-tagged self-complementary adeno-associated viral vector 2 (scAAV2) driven by a murine RPE65 promoter. Enhancing RAP1a activity specifically in RPE cells stimulated barrier integrity, inhibited choroidal endothelial cell transmigration in the RPE monolayer, and suppressed choroidal neovascularization without causing any functional or morphological deficits in the retina. These positive effects of RPE-specific RAP1 activation were attributed to protection of RPE junctional complexes by IQGAP-1 (IQ Motif Containing GTPase Activating Protein 1). IQGAP-1 interacts with β-catenin and aids the formation of junctional Cadherin/β-catenin complexes, thereby contributing to RPE junctional integrity [70]. Results from a similar study revealed that active RAP1 prevents the development of choroidal neovascularization via the following mechanisms: (a) inhibition of NADPH-oxidase dependent Rac1 and NF-κB activation, (b) impairment of ROS-dependent signaling, and (c) suppression of TNF-α-induced choroidal endothelial cell migration [71]. Therefore, based upon these findings, RAP1 may be a potential clinical therapeutic candidate for neovascular AMD (Figure 4).

### 2.5. Celecoxib

Immunologic responses such as inflammatory cytokines, macrophages, activation of complement and microglia play crucial roles in AMD pathology [72]. Differential expression of complement markers has been linked to damaged AMD mitochondria and cells [73]. Prostaglandins (PG) play a key role in triggering the inflammatory responses. Cyclooxygenase (COX) enzymes catalyze the biosynthesis of proinflammatory prostaglandins. In the eye, prostaglandins are released in response to injury and affect retinal blood flow, induce VEGF, increase vascular permeability and leukocyte migration, thereby contributing to intraocular inflammation and breakdown of the blood–ocular barrier (i.e., blood–retinal and blood–aqueous barriers) [74,75]. COX-2, the primary inducible COX isoform found in human RPE cells, is predominantly involved in inflammatory responses, and induced by inflammatory cytokines and pathogenic stimuli such as bacterial LPS (lipopolysaccharide). COX-2 induction contributes to both physiologic and pathologic conditions in the RPE. COX-2 is expressed in human neovascular membranes and contributes to AMD choroidal neovascularization [76].

**Figure 5:**
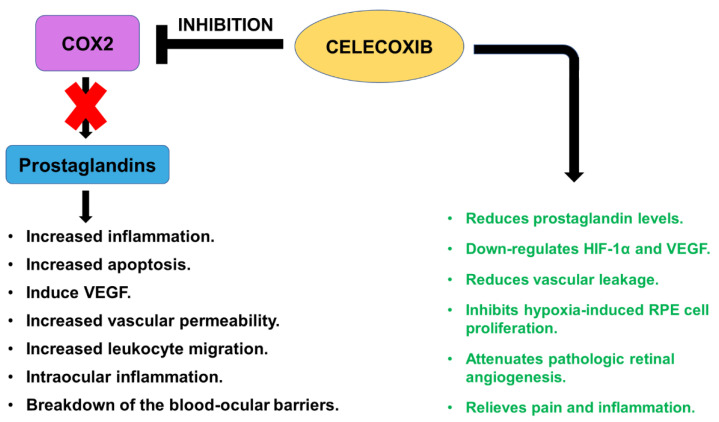
Celecoxib’s mechanism of action. Celecoxib inhibits COX2 (Cyclooxygenase-2) and prevents the pro-apoptotic and pro-inflammatory action of prostaglandins. Celecoxib reduces prostaglandin levels, down-regulates HIF-1α and VEGF, reduces vascular leakage, inhibits hypoxia-induced RPE cell proliferation, attenuates pathologic retinal angiogenesis, and relieves pain and inflammation.

Inhibition of COX-2 reduces ocular angiogenesis and may be useful for the treatment and prophylaxis of AMD pathology [77]. Various topical ocular NSAIDs (Non-Steroidal Anti-Inflammatory Drugs) are routinely used to reduce intraocular inflammation after vitreoretinal and cataract surgery. Celecoxib is a benzene sulfonamide NSAID that is widely used to relieve pain and inflammation in diseases such as arthritis, ischemia, seizures, and acute pain. Celecoxib’s primary mechanism of action is selective inhibition of COX-2 enzyme. Celecoxib is several-fold more potent in inhibiting COX-2 compared to COX-1. In the eye, Celecoxib’s rescue effects have been well-established. Celecoxib down-regulates HIF-1α and VEGF via the angiogenesis-associated PI3K/AKT-dependent pathway and inhibits hypoxia-induced RPE cell proliferation in vitro [78,79]. Previous in vitro studies with ARPE-19 cells have demonstrated that Celecoxib in nanomolar concentrations inhibits VEGF at both transcript and protein levels. No cytotoxicity was detected at VEGF-inhibiting concentrations of Celecoxib [80]. Oral administration of Celecoxib inhibits COX-2, reducing VEGF levels and vascular leakage in vivo [81]. However, the caveats of the oral delivery route are: (1) since COX is also present in other body tissues, frequent oral consumption of this drug also causes systemic side-effects; and (2) higher drug dose is required to allow sufficient amounts to reach the eye. To this end, the effects of periocular injections of Celecoxib were evaluated in vivo. Celecoxib-PLGA (Poly Lactic-co-Glycolic Acid) microparticles were administered via subconjunctival injection in a streptozotocin-diabetic Sprague Dawley rat model and this caused sustained drug delivery to the retina that led to higher retinal drug levels, reduced VEGF expression, and lowered vascular leakage [82,83]. In a similar study, Kim et al. demonstrated that intravitreal injection of Celecoxib provided prolonged drug delivery in rabbit eyes for up to 8 weeks. Celecoxib reduced inflammation and PG levels without causing toxicity [84]. Its low solubility in vitreous allows prolonged absorption, giving Celecoxib an extended effective intraocular half-life [85]. Since Celecoxib exhibits anti-inflammatory, anti-angiogenic, and anti-proliferative properties in the retina, CELEBREX^®^ i.e., a celecoxib prescription drug, is currently in clinical trials and being tested in neovascular wet AMD patients undergoing photodynamic therapy (Figure 5).

### 2.6. SS-31/Elamipretide

Healthy mitochondria ensure proper functioning of key cellular bioenergetic and metabolic pathways thereby contributing to cellular and physiological homeostasis [83]. Substantial evidence implicates dysfunctional mitochondria in the etiology of AMD [86]. Our previous studies demonstrated significant mitochondrial toxicity as evidenced by increased mtDNA fragmentation, reduced mtDNA copy number, abnormal OXPHOS complex protein subunits’ levels, and aberrant expression of mitochondrial transcription and replication genes in human AMD cybrid RPE cell lines where mitochondria are derived from AMD patients [12,13]. Mitochondria are the major sources of energy, i.e., adenosine triphosphate (ATP) in the cells and damaged mitochondria lead to ATP depletion. Decreased oxygen levels due to hypoxia and ischemia result in loss of mitochondrial cristae membranes thereby compromising ATP recovery which is essential to minimize tissue damage. Mitochondrial ATP synthesis requires intact mitochondrial cristae which are invaginations of the inner mitochondrial membrane. Cardiolipin, an anionic diphosphotidylglycerol lipid synthesized and predominantly found in the inner mitochondrial membrane, is required for mitochondrial cristae formation and efficient oligomeric assembly of electron transport chain complexes. Cardiolipin’s structural motif is composed of four acyl chains linked to two phosphate groups and a glycerol moiety. Cardiolipin is exclusively localized in the mitochondrial membranes in mammalian mitochondria where it stabilizes the inner membrane proteins [87]. It enables optimal ATP production as it functions as a proton trap for ATP synthase and facilitates electron transfer by electrostatic interaction with cytochrome c. Cardiolipin enhances the peroxidase activity of cytochrome c, which results in higher levels of oxidized cardiolipin. Cardiolipin is also involved in the mitochondrial fission and fusion processes. Therefore, Cardiolipin is vital in normal mitochondrial functioning and its deficiency disrupts the respiratory complexes, increases proton leak, enhances ROS production, impairs ATP synthesis, alters mitochondrial morphology, and subsequently compromises mitochondrial dynamics and stability. Moreover, aging alters cardiolipin’s lipid composition, thereby affecting mitochondrial membrane structure and causing destabilization of respiratory complexes in aged tissues [88,89]. Decline in cellular ATP increases mitochondrial calcium levels, inducing cardiolipin peroxidation and opening of the Mitochondrial Permeability Transition (MPT) pore, leading to ROS generation, and release of cytochrome c from the mitochondria to the cytosol [90].

In recent years, pharmacological strategies that target inner mitochondrial membrane and cardiolipin have been developed. SS-31 is a water-soluble, mitochondria-targeting antioxidant that belongs to a family of Szeto–Schiller (SS) peptides named after Hazel H. Szeto who created these SS peptides in collaboration with Peter W. Schiller. The SS peptides inhibit oxidative cell death with EC_50_ in the nanomolar range. The free radical scavenging property of SS peptides can be ascribed to dimethyltyrosine or tyrosine residues that scavenge oxyradicals, thereby forming relatively unreactive tyrosyl radicals. SS-31 is also known as Mitochondria Targeting Peptide-131 (MTP-131), Elamipretide, and Bendavia. SS-31 is a cell permeable, aromatic–cationic tetrapeptide with a molecular weight of 639.8 g/mol. Its chemical structure is H-d-arginyl-2,6-dimethyl-l-tyrosyl-l-lysyl-l-phenylalaninamide, and its structural motif is formed by aromatic residues and basic amino acids. At physiologic pH, SS-31 carries a 3+ net charge, and it selectively targets and concentrates in the inner mitochondrial membrane, by interacting with cardiolipin. By targeting the inner mitochondrial membrane, SS-31 scavenges ROS at their site of production and reduces mitochondrial ROS. In response to calcium overload, SS-31 inhibits MPT and minimizes MPT-induced ROS accumulation, prevents mitochondrial swelling, reduces cytochrome c release, and inhibits cytochrome c peroxidase activity. Moreover, SS-31 scavenges the oxidants, i.e., hydrogen peroxide and peroxynitrite ions, and prevents oxidation of linoleic acid and low-density lipoprotein [91].

SS-31 exerts antioxidant and anti-apoptotic effects in both in vitro and in vivo models of neurogeneration, ischemia–reperfusion injury, cardiac diseases, and insulin resistance. Preclinical studies have shown that SS-31 improves retinal function as demonstrated by reduction in ERG a-wave and b-wave amplitudes. Moreover, SS-31 reduced TUNEL-positive cells, downregulated BAX, inhibited the release of cytochrome c, and suppressed mitochondrial dysfunction in the retina. Treatment with SS-31 lowered levels of retinal superoxide dismutase 2 (SOD2), an antioxidant, and Malondialdehyde (MDA), an oxidative stress marker and product of cellular fatty acid peroxidation [92]. Another study demonstrated SS-31-mediated significant cytoprotection against chronic oxidative stress induced by hydrogen peroxide in ocular cell lines. This study revealed that pretreatment with SS-31 prevented mitochondrial depolarization, reduced intracellular ROS levels and LDH release, alleviated apoptosis, inhibited cytochrome c release from the mitochondria to the cytoplasm, suppressed Caspase-3 activation, and prevented cytoskeleton disarrangement, thereby maintaining cellular integrity under hydrogen peroxide-induced sustained oxidative stress [93]. Furthermore, SS-31 minimizes the effects of ischemia–reperfusion injury by accelerating ATP recovery.

**Figure 6:**
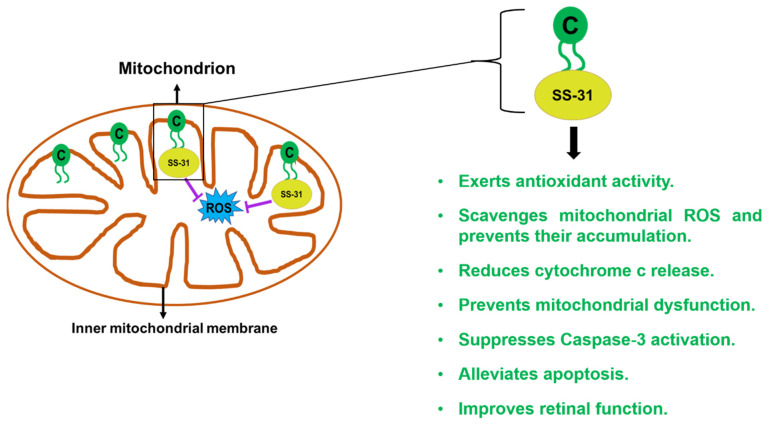
SS-31/Elamipretide’s mechanism of action. SS-31 exerts its protective effects by binding to Cardiolipin on the inner mitochondrial membrane. SS-31 bound to Cardiolipin prevents the accumulation of ROS, exerts antioxidant effects, reduces apoptosis, prevents mitochondrial dysfunction, and promotes retinal function.

Recently, to evaluate the safety and tolerability of subcutaneous SS-31/Elamipretide in subjects with intermediate AMD, a Phase 1 single-center clinical trial was sponsored by Stealth BioTherapeutics [94]. This was an open-label, Phase 1 clinical study in approximately 40 subjects who had 1 eye with intermediate AMD. Subgroups included some subjects with high-risk drusen without geographic atrophy and others with noncentral geographic atrophy. All study subjects received 40 mg of Elamipretide administered as a once daily 1.0 mL subcutaneous injection for 12 weeks. Currently, Elamipretide is being investigated by Stealth BioTherapeutics in a Phase 2, randomized, double-masked, placebo controlled clinical trial to evaluate the safety, efficacy and pharmacokinetics of subcutaneous injections of Elamipretide in approximately 180 patients with dry AMD and geographic atrophy. The endpoints will be safety and tolerability, low-luminescence best-corrected visual acuity, geographic atrophy area measured by OCT and fundus autofluorescence (Figure 6).

## 3. Conclusions and Future Directions

In conclusion, the above-mentioned therapeutic candidates that target various aspects of AMD etiology are already either in preclinical or clinical development and will await the outcomes of the clinical trials for establishment of therapeutic potential in humans.

With the advent of precision medicine and genetic engineering tools, the future for other untapped novel therapeutics seems promising but challenging. More recently, CRISPR as a cutting-edge, genome editing technology, has been propelled to the forefront of translational ocular research and has been successfully applied to rescue retinal cells in various retinal degenerative diseases both in vitro and in vivo. The CRISPR gene editing technique can be further tweaked for development of new therapies for AMD. Variants of the canonical CRISPRCas9 system such as CRISPR interference (CRISPRi) and CRISPR activation (CRISPRa) techniques allow transcriptional silencing and activation of genes, respectively, and provide an advantage of reversibility in CRISPR gene editing.

In the quest for a cure for AMD, further research will entail examining the additive effects of combination therapies in pre-clinical models of AMD and advancement of groundbreaking bench research to clinical trials.
